# Anterior Access to the Cervicothoracic Junction via Partial Sternotomy: A Clinical Series Reporting on Technical Feasibility, Postoperative Morbidity, and Early Surgical Outcome

**DOI:** 10.3390/jcm12124107

**Published:** 2023-06-17

**Authors:** Mohammed Issa, Jan-Oliver Neumann, Sameer Al-Maisary, Gerhard Dyckhoff, Moritz Kronlage, Karl L. Kiening, Basem Ishak, Andreas W. Unterberg, Moritz Scherer

**Affiliations:** 1 Department of Neurosurgery, Heidelberg University Hospital, 69120 Heidelberg, Germany; 2 Department of Cardiac Surgery, Heidelberg University Hospital, 69120 Heidelberg, Germany; 3 Department of Otorhinolaryngology, Heidelberg University Hospital, 69120 Heidelberg, Germany; 4 Department of Neuroradiology, Heidelberg University Hospital, 69120 Heidelberg, Germany

**Keywords:** cervicothoracic junction, anterior thoracic corpectomy, partial sternotomy

## Abstract

Surgical access to the cervicothoracic junction (CTJ) is challenging. The aim of this study was to assess technical feasibility, early morbidity, and outcome in patients undergoing anterior access to the CTJ via partial sternotomy. Consecutive cases with CTJ pathology treated via anterior access and partial sternotomy at a single academic center from 2017 to 2022 were retrospectively reviewed. Clinical data, perioperative imaging, and outcome were assessed with regards to the aims of the study. A total of eight cases were analyzed: four (50%) bone metastases, one (12.5%) traumatic instable fracture (B3-AO-Fracture), one (12.5%) thoracic disc herniation with spinal cord compression, and two (25%) infectious pathologic fractures from tuberculosis and spondylodiscitis. The median age was 49.9 years (range: 22–74 y), with a 75% male preponderance. The median Spinal Instability Neoplastic Score (SINS) was 14.5 (IQR: 5; range: 9–16), indicating a high degree of instability in treated cases. Four cases (50%) underwent additional posterior instrumentation. All surgical procedures were performed uneventfully, with no intraoperative complications. The median length of hospital stay was 11.5 days (IQR: 9; range: 6–20), including a median of 1 day in an intensive care unit (ICU). Two cases developed postoperative dysphagia related to stretching and temporary dysfunction of the recurrent laryngeal nerve. Both cases completely recovered at 3 months follow-up. No in-hospital mortality was observed. The radiological outcome was unremarkable in all cases, with no case of implant failure. One case died due to the underlying disease during follow-up. The median follow-up was 2.6 months (IQR: 23.8; range: 1–45.7 months). Our series indicates that the anterior approach to the cervicothoracic junction and upper thoracic spine via partial sternotomy can be considered an effective option for treatment of anterior spinal pathologies, exhibiting a reasonable safety profile. Careful case selection is essential to adequately balance clinical benefits and surgical invasiveness for these procedures.

## 1. Introduction

The cervicothoracic junction (CTJ) and upper thoracic spine are challenging regions for surgical accessibility, particularly when anterior access is required for spine pathology affecting the anterior column or vertebral bodies [1,2]. Bone metastases, primary bone neoplasms, traumatic or osteoporotic fractures, spondylodiscitis, and disc prolapses are common pathologies affecting the vertebral bodies, frequently compromising spine stability in this area. In such cases, surgical therapy is often required to restore stability of the CTJ and upper thoracic spine [3]. In the literature, there are controversial opinions as to whether an anterior or posterior approach is most favorable to access this area of the spine [4,5,6,7,8,9,10,11,12]. The posterior or posterior-lateral approach to the upper thoracic spine and the CTJ involve manipulation of the spinal cord [13,14] to reach the vertebral bodies and carry a pronounced risk for postoperative morbidity associated with these extended approaches [3,14]. Particularly, wound-healing disorders and infections are reported to occur in up to 30% of cases [13,15].

In anterior approaches to the CTJ, the transition from cervical lordosis to thoracic kyphosis is a challenge as the vertebral bodies turn away from the field of view [8]. Partial sternotomy, as an alternative approach, allows reasonable exposure of the vertebral bodies; however, little is known about early and late morbidity of this presumably invasive access route [10,16,17]. While the normal median sternotomy is associated with thoracic instability, significant pain intensity, postoperative infections, and wound-healing disorders, the minimal partial sternotomy, limiting bone cuts to the third intercostal recess, offers a good alternative with significantly lower rates of the aforementioned complications [18].

This study provides a detailed evaluation of the anterior approach via partial sternotomy for anterior access to the CTJ region in terms of technical feasibility, postoperative morbidity, and early surgical outcome.

## 2. Materials and Methods

### 2.1. Study Design and Patient Population

This is a retrospective evaluation of a consecutive clinical series of cases who underwent a corpectomy in the CTJ region due to pathologies in the anterior structures through anterior access via partial sternotomy between August 2017 and May 2022. The institutional review board consented to the conduction of this study, and the requirement for informed patient consent was waived for this retrospective series (Ref S-723/2017).

### 2.2. Outcome Parameters 

Clinical parameters, such as sex, age, American society of anesthesiologist (ASA) score, hospital and intensive care unit (ICU) stay, intraoperative blood loss, and necessity of blood transfusion, were evaluated. The instability of the spine was assessed preoperatively based on the Spinal Instability Neoplastic Score (SINS) [19]. The SINS was calculated for each patient to classify spinal instability and grouped as either stable (SINS = 1–6), potentially instable (SINS = 7–12), or instable (SINS = 13–18). Oncologic prognosis was estimated by the Tokuhashi score to guide surgical decision making in oncologic cases. Clinical outcome was assessed by medical chart review with regards to pre- and post-operative neurological status, need for transfusion, need for pain killers, morbidity, and mortality during hospital stay and during follow-up, respectively. 

### 2.3. Radiological Outcome

The diagnosis of the pathology in the upper thoracic and cervicothoracic junction was based on magnetic resonance imaging (MRI) and computed tomography (CT) scans. Routine clinical and radiological follow-up examinations were performed before discharge. Standard X-ray and CT scans encompassing the anteroposterior and lateral view were performed to evaluate cage position and fusion rate.

### 2.4. Surgical Technique

All surgical procedures in this series were performed using a combination of neurosurgery, cardiovascular surgery, otorhinolaryngology, and head and neck surgery at an academic center. A thoracic vertebral body corpectomy was performed in all eight patients using the anterior access via partial sternotomy. Before placing the patient in a supine position and fixing the head into the Mayfield holder, all patients received endotracheal anesthesia and neuromonitoring of the recurrent laryngeal nerve through use of the advanced laryngeal monitoring tube (ALM tube) (inomed, Emmendingen, Germany). Using intraoperative fluoroscopy, the level of the pathology was localized. Cervical access was chosen on the patient’s right side.

**Figure 1:**
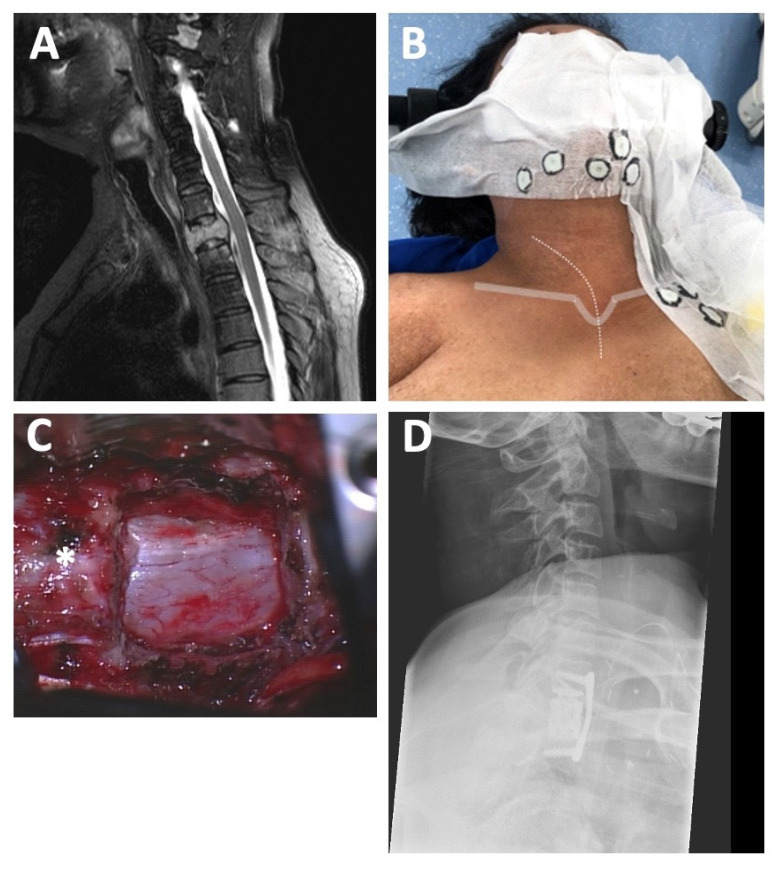
Spinal metastases. (**A**) Preoperative MRI of the CTJ showing a metastatic osteolytic lesion of T1 with an accompanying pathologic fracture (SINS 16). (**B**) Intraoperative landmarks for anterior access: skin incision (dotted grey line) starting at the level of the larynx follows the medial border of the sternocleidomastoid muscle towards the jugulum (solid grey line) and extends to the upper third of the sternum medially. CT-opaque fiducials (circled in black) were used for navigation guidance in this case using an intraoperative CT scan of the cervicothoracic junction. (**C**) Intraoperative microscopic view after complete T1 corpectomy and tumor removal with wide exposition of the dura (* indicates C7 vertebra). (**D**) Postoperative X-ray illustrating excellent implant position after standalone anterior treatment.

Figure 1B illustrates the skin incision, median of the upper sternum, and anterior margin of right sternocleidomastoid. The use of navigation was confirmed at the discretion of the surgeon based on intraoperative CT scans. CT-visible fiducials were glued for marking (Figure 1). After transection of the platysma and exposure of the sternum and upper cervical fascia, the otolaryngologist performed cervical dissection to expose the cervical spine. In common blunt interfacial dissection, the vascular nerve sheath, along with the carotid artery and jugular vein, is exposed and lateralized and the dissection is deepened to the prevertebral fascia. Then, dissection is extended caudally on the prevertebral fascia as low as possible with continuous monitoring of the right recurrent laryngeal nerve. Partial sternotomy is performed by the cardiac surgeon from the cranial border of the sternum into the third intercostal space on the right, and the sternum is distracted to widen access. Visualization of the brachiocephalic vein and palpation of the position of the aortic arch are caudal limits of the exposure. As a final step of the exposure, cervical and sternal approaches must be combined in the area we refer to as no man’s land.

**Figure 2:**
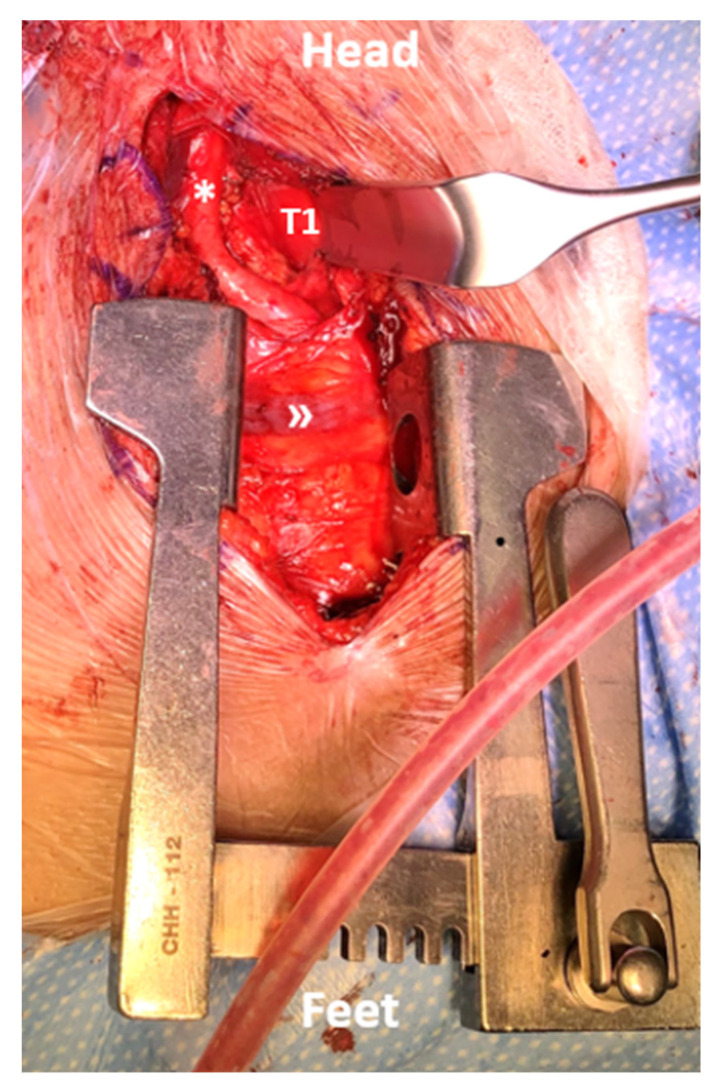
Intraoperative view of the distraction phase and exposure of the mediastinum for an anterior T1 corpectomy. The proximal carotid artery (*) and brachiocephalic trunk (») are visualized boundaries of the approach.

In this area, with which the surgical specialists involved are usually not familiar, sharp transection is needed on the sterno-thyroid muscles for mobilization of the larynx medially and inter-fascial dissection is deepened to retract the carotid artery and jugular veins laterally. The course of the recurrent laryngeal nerve is carefully monitored at this point to prevent stretching injury. Depending on the segment addressed, ligation of thyroid vessels can additionally be required. Soft tissue retraction is achieved by common cervical retractors (Jarit, Integra, Princeton, NJ, USA), and the correct exposure is verified by a.p. and lateral fluoroscopy. Figure 2 illustrates the view of the anterior procedure of the approach.

Once exposure of the desired level is completed, surgery is continued with neurosurgery as required for the pathology treated. In all cases in this series, anterior corpectomy was performed by incision of the disc spaces and resection of the vertebral bodies using standard techniques. Subsequently, decompression or tumor resection was continued as needed under the operation microscope. After the superior endplate of the lower vertebral body and the inferior endplate of the upper vertebral body had been exposed and prepared, a distractable cervical spine cage (Xpand, Globus Medical, Audubon USA or obelisc, ulrich medical, Ulm, Germany) was inserted and extended, and its position was checked by an anterior-posterior and lateral X-ray. Depending on the surgical concept, an additional anterior plating or dorsal instrumentation was performed to ensure stability of the spine, at the discretion of the surgeon. Closure of the cervical exposure was limited to the re-adaptation of sterno-thyroid muscles and the platysma. Closure of the sternum was achieved by standard wire cerclage in this series. A deep drainage (10 Char) was positioned at the anterior vertebral levels and removed on postoperative day two. Wound closure was achieved by intracutaneous stitching in this study. Postoperative pain regimen included one NSAR- and Opioid-Painkiller on POD 1–3. 

Physical therapy was initiated on the first day after surgery. The sternum is regarded as fully resilient again 3 months after partial sternotomy. Until then, patients should not carry heavy objects more than 5 kg. In addition, work and sports that put a strain on the sternum should be avoided. 

### 2.5. Statistical Analysis

Normal distribution was tested using the Shapiro–Wilk test. Continuous variables were reported as median, interquartile range (IQR) and categorical variables as frequencies and percentages. A *p*-value of less than 0.05 was regarded as a statistically significant difference and was determined by the t-test for continuous variables and the chi-squared test for nominal variables. Statistical analyses were performed using SPSS 27 (IBM Corp., Armonk, NY, USA).

## 3. Results

### 3.1. Patient Characteristics 

This retrospective case series encompasses a total of eight cases with an acute pathology of the CTJ/upper thoracic spine from C7 to T4 undergoing anterior thoracic corpectomy and fusion via partial sternotomy between August 2017 and May 2022. Pathologies included were four (50%) pathologic fractures due to bone metastasis, one (12.5%) traumatic instable chance fracture (B3-AO-Fracture) after a bicycle accident, one (12.5%) thoracic disc herniation with spinal cord compression, and two (25%) infectious pathologic fractures from tuberculosis and spondylodiscitis, respectively. The median age was 49.9 years (IQR: 21; range: 22–74 years); six (75%) were male, and two (25%) were female. Six (75%) cases were classified as American Society of Anesthesiologists (ASA) grade 2, and two (25%) cases were classified as ASA grade 3. The median Spinal Instability Neoplastic Score (SINS) was 14.5 (range: 9–16). Accordingly, three (37.5%) potentially unstable cases (9, 11, and 12 points in the SINS, respectively) presented with an unstable AO-B3 fracture, ossified disc herniation with massive spinal cord compression, and spondylodiscitis with incipient anterior column tilting, respectively; five (62.5%) unstable cases (14, 15, and 3 × 16 points in the SINS, respectively) presented with subluxation or translation and >50% collapse of the vertebral bodies in all five cases and bilateral posterolateral involvement of the spinal elements in three cases. The four metastasis cases showed a median Tokuhashi score of 9 points (IQR: 3; range: 9–13), implying a survival prognosis of more than 6 months and thus supporting an excision [20].

All patients suffered preoperative axial loading pain in the cervicothoracic transition region correlating to pending instability, three patients had dermatome-related radiculopathy symptoms, one patient presented with severe arm paralysis, and one presented with spinal ataxia. Detailed patient characteristics are described in Table 1.

### 3.2. Surgical Outcomes and Postoperative Complications

All procedures were performed via a right-sided approach under stimulation-monitoring of the recurrent laryngeal nerve. Stimulation could locate the nerve in all cases in this study. Four (50%) cases with a SINS of more than 14 points and high kyphosis in the CTJ underwent additional posterior instrumentation; the other four (50%) cases had standalone anterior fusion. 

All surgical procedures were performed uneventfully with no new additional postoperative neurological deficits. The median length of hospital stay was 11.5 days (range: 6–20 days), including a median of 2.6 days in ICU (range: 0–20 days). Blood transfusion was necessary in four cases. The median blood loss was 550 (range: 200–1700 mL). The median duration of the procedure was 290 min (range: 241–515 min). Postoperative thyroid function was unremarkable according to laboratory testing.

Two (25%) cases developed postoperative dysphagia with a maintained unrestricted oral diet. This was related to a transient dysfunction of the recurrent laryngeal nerve with hoarseness but without dyspnea. Both cases were closely monitored by otolaryngology, and recovery was assisted by logopedic training. At 3 months follow-up, both cases showed complete recovery, indicating a temporary dysfunction caused by stretching the recurrent laryngeal nerve during surgery.

Two (25%) cases developed a wound-healing disorder affecting the posterior approach, requiring surgical revision. There was no sternal instability or post-sternotomy pain in our cohort. All cases were discharged on non-steroidal pain medication only. No in-hospital mortality was observed.

One case died due to the underlying disease during follow-up. The median follow-up was 2.6 months (average: 1–45.7 months).

Surgical outcome is summarized in Table 2. 

### 3.3. Radiological Findings

Postoperative conventional X-ray images in lateral and anterior-posterior projection and CT scans were obtained in each case to confirm stability of the instrumentation. The radiological outcome was unremarkable in all cases, with no case of implant failure between early postoperative imaging and imaging at the last follow-up.

### 3.4. Case Illustration 1: Spinal Metastases

A 43-year-old female with a history of breast cancer presented at our department with increasing localized cervical and thoracic pain deteriorating with axial loading. The neurological examination revealed left-sided hypesthesia in the supply area of the C8 nerve root. In the subsequent full-spine CT and MRI imaging, a metastatic osteolytic lesion with paravertebral tumor involvement (T1) with an accompanying pathologic fracture was observed, resulting in a SINS of 16 with more than 50% collapse of the vertebral body and massive kyphosis of the CTJ, indicating spinal instability (Figure 1A).

Due to the underlying condition, the patient was given an ASA score of three, which led to the choice of an anterior access route for T1 corpectomy, tumor debulking, and fusion in a standalone concept in this patient. Anterior fusion from C7 to T2 without posterior fusion of the CTJ was achieved in this case. Postoperatively, the patient recovered completely from the C8 hypesthesia and loading pain and was discharged home after one day in the ICU and a total of 8 days in the hospital. The adjuvant chemotherapy and local radiation of the spine could begin three weeks after surgery. Despite the favorable initial course, the patient died from further systemic hepatic and pulmonary metastatic disease one year after the procedure.

Figure 1 shows representative pre- and post-operative images of this case as well as landmarks for skin incision.

### 3.5. Case Illustration 2: Junctional Disc Herniation

**Figure 3:**
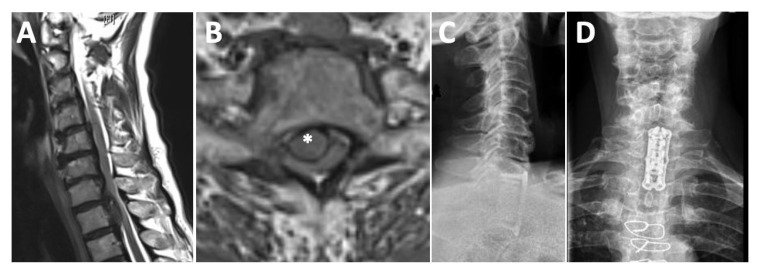
Junctional disc herniation. Preoperative T2-weighted MRI in sagittal (**A**) and axial (**B**) orientation showing a large C7/T1 central disc herniation with caudal extension to T2, severe spinal cord compression, and acute myelopathy (herniated disk is highlighted by * in (**B**)). Postoperative X-ray in lateral (**C**) and anterior-posterior (**D**) view, confirming excellent implant position after standalone anterior treatment.

A 74-year-old female patient presented with a 2-month history of progressive spinal ataxia of the lower extremities, midback pain, and myelopathic signs with decreased sensation distributed below T2. After lifting a heavy weight, symptoms deteriorated. MRI revealed a C7/T1 central disc herniation with severe spinal cord compression and acute myelopathy (Figure 2A,B). The CT showed extended calcifications of the herniated disc sub-totally occupying the spinal canal. The patient was treated via an anterior approach with partial sternotomy, C7 and T1 corpectomy, and microsurgical decompression of the spinal canal. Vertebra replacement was achieved with an expandable cage and anterior plating in a standalone concept. Postoperatively, the patient experienced immediate relief of her myelopathic symptoms and was able to walk without assistance by postoperative day 7. At 3 months follow-up, the patient reported complete symptom relief with no residual back pain. Figure 3 shows the preoperative MRI and the postoperative X-ray.

## 4. Discussion

The anterior access to the cervicothoracic junction and upper thoracic spine via partial sternotomy or sole manubriotomy has been previously described in cadaveric studies and several clinical studies [21,22,23,24]. The regional anatomy of the CTJ (including the aortic arch caudally), the underlying pathologies requiring surgical treatment, and the location at the interface of different surgical disciplines are challenging aspects of this approach which bring a risk of intra- and post-operative morbidity [2,8,9,11,14]. In our series of eight consecutive cases, we could illustrate that this approach warrants safe and effective access to the CTJ in an interdisciplinary effort with the use of contemporary neuromonitoring techniques. In this discussion, we would like to reflect on the tenets and techniques to handle the anterior access to the CTJ and emphasize diligent case selection to balance risks and benefits of the procedure.

Even though ventral access to the cervical spine is among the standard approaches in spine surgery, lowering this approach to the CTJ is challenging from various perspectives. Particularly, traversing thyroid vessels and the recurrent laryngeal nerve can restrain access cranially. Caudally, the sternum and the large subclavian and jugular junctions limit the exposure. In our multidisciplinary approach, neuromonitoring of the recurrent laryngeal nerve enabled safe identification of the nerve in all cases. This enabled exposure and careful mobilization of the nerve during the procedure. Nevertheless, stretching the nerve can cause temporary dysfunction with unilateral vocal cord palsy, which was observed in two (25%) cases in our study. In the literature, this complication is reported in up to 24% of cases following an anterior cervical discectomy and fusion (ACDF) and generally shows good potential for improvement [25]. Both adverse events were closely monitored and showed complete recovery, indicating that no permanent damage was caused to the recurrent laryngeal nerve. However, dysphagia and temporary hoarseness must be considered as typical complications of the anterior access and must be taken into account when evaluating access routes to the CTJ. 

Partial sternotomy allows for adequate exposure of the CTJ without destabilizing the thorax. This has been shown to have various benefits over conventional sternotomy with regards to recovery, wound-healing disorders, and postoperative pain [18]. Moreover, anterior bracing of the thorax remains in support of the thoracic spine even after this procedure, which should be considered for surgery of the CTJ. 

Despite combining three surgical specialties for this approach, combining the deep aspects of the cervical and thoracic approaches leads to an area we refer to as no man’s land. We observed that this step of the exposure is not familiar to any involved specialty and thus requires collaboration to handle large vessel exposure and cervical soft-tissue mobilization to expose the anterior aspect of the CTJ.

Regarding overall morbidity, we did not observe significant procedure-related morbidity despite the cases of dysphagia mentioned above. Particularly, we did not observe any complications related to sternotomy with regards to sternal instability, wound-healing disorders, or chronic pain states [8,10,18].

The selection of the approach is always key in complex spine pathology. All cases presented with disease affecting the CTJ and showed clear evidence of spinal instability according to the SINS score, primarily arising from the anterior column (median SINS: 14.5; range: 9–16). Under evaluation of other possible approaches and surgical concepts, an anterior access was chosen for all cases. In our retrospective settings, the choice for this approach was not controlled, but Tokuhashi scores (median: 9; range: 9–13) suggested a fair prognosis, thus warranting this approach in all cases.

De Giacomo et al. included 142 cases with anterior surgery of the thoracic spine in their 2011 study [26]: cervico-sternal split surgery (15), thoracotomy (85), video-assisted thoracoscopic surgery (VATS) (10), and thoracolumbar surgery (32). Overall, 21.8% of the patients showed postoperative complications; the cervicothoracic approach, using a partial sternotomy, showed the lowest complication rate at 6.7% compared to the other approaches [26]. This complication rate is comparable with our series, which includes two cases of temporary paresis of the recurrent laryngeal nerve (25%). In our cases, we had two posterior wound infections, which were associated with the posterior vertebral body fusion (2/4; 50%). This is comparable with the existing literature, where a wound-healing disorder rate of up to 30% in the posterior approach to the CTJ has been reported [13,15].

Brogna and colleagues [10] performed an anterior approach to the CTJ in 18 patients over the course of five years. Comparably to our study, no exposure-related morbidity was reported intraoperatively. However, a significantly higher mean rate of blood loss was reported (800 mL) compared to the mean in our series (550 mL). As observed in our cohort, variation of blood loss is also dependent on the underlying pathology at the vertebra. As the majority of the anterior exposure is achieved by interfacial dissection, sternotomy is usually the only source of relevant bleeding during the exposure. Here, hemostasis can usually be achieved by monopolar cautery or by applying bone wax.

The question of whether to perform an additional posterior stabilization after an anterior approach is handled very heterogeneously in the literature. In the series by Brogna et al., 30% of cases needed additional posterior fixation in comparison to 50% of cases in our series [10]. In our series, the decision for additional posterior instrumentation was made at the discretion of the surgeon, depending on the initial degree of instability and according to intraoperative findings. In three oncologic cases, challenges to providing solid anterior plating led to additional posterior fixation, and one traumatic type-c luxation fracture required 360° fusion (Table 1). Particularly in oncologic cases, however, individual decision making should identify cases suitable for a standalone anterior procedure because advantages of the anterior exposure are expected to be greatest when used as an alternative rather than an addition to a posterior approach. However, the patients who were additionally operated on from the posterior approach in our series had a longer hospital stay.

The side of access to the CTJ has been chosen heterogeneously in previous studies. Kraus et al. chose the left posterior-superior mediastinum in close approximation to the vertebral bodies of T1 and T2 [11]. Other authors make the side of access patient-dependent, such as Prezerokos et al., and describe both sides of the thoracotomy as possible approaches to the CTJ [9]. Nonetheless, most surgeons opt for the right-sided sternotomy approach. Besides familiarization from anterior cervical discectomy and fusion (ACDF) approaches, the advantage of a right-sided approach is the avoidance of the aortic arch, which is an anatomic limitation of the anterior exposure of the CTJ [8,10,26,27,28]. In addition, the left recurrent nerve shows a longer course over the aortic arch and is therefore more tangible during the exposure in a left-sided approach [29].

In summary, anterior access via partial sternotomy should be considered a viable option in cases of anterior spine pathology in the CTJ region. Under careful case selection, the rates of reported complications are tolerable and must be weighed against the morbidity that has to be expected from a posterolateral approach as an alternative [10,11,26,30]. Even though the term ‘sternotomy’ reasonably triggers reservations as it suggests an invasive procedure, we aimed at illustrating that exposure predominantly involves blunt interfascial cervical dissection, with the sternothyroid muscles being the only structure that needs to be cut along the way. In addition, partial sternotomy, as performed in our series, has the advantage of maintaining sternal stability and thus of mitigating the majority of complications usually associated with this procedure.

In our opinion, and despite our own initial reservations, anterior access should be considered as an option in anterior column disease of the CTJ, particularly when intact posterolateral structures could enable a standalone anterior treatment. One main advantage of this concept is the potential to spare on posterior approaches to the CTJ known to be associated with significant morbidity throughout [13,15].

### Limitation: Selection

The findings of this study should be interpreted within the scope of its limitations. Both the retrospective design and the small sample size affect the generalizability of the results. It must be acknowledged that outcomes presented in this series result from a highly selected cohort of heterogeneous pathology of the CTJ with promising prognosis according to a high Tokuhashi score. The benefits to address the anterior spinal column via an anterior approach must be well balanced against the risks of the procedure, and the decisions must be made under careful consideration of overall patient prognosis regarding the underlying disease. Diligent selection of suitable cases for an anterior approach and sternotomy is a mainstay for satisfactory results reported in this series.

Furthermore, performance bias must be considered in the context of the complex procedure evaluated in this series. As these cases were performed at an academic neurosurgical center with an experienced and familiarized interdisciplinary team, reported outcomes may vary significantly in other centers and setups. Additionally, our follow-up was able to show good surgical outcome at the early stage. However, a longer follow-up period is necessary to uncover late morbidity and to evaluate the effect of surgery on oncologic outcome in more detail.

The paucity of existing evidence and heterogeneity of reported cohorts and treatment approaches set significant barriers to our clinical findings and prevents from drawing robust conclusions. Consequently, treatment decisions towards anterior access to the CTJ must be made on an individually balanced basis. Longer follow-up periods and larger cohorts are essential to extrapolate definite outcomes and results of the approach.

## 5. Conclusions

Our series indicates that the anterior approach to the cervicothoracic junction and upper thoracic spine via partial sternotomy can be considered an effective option for treatment of anterior spinal pathologies, exhibiting a reasonable safety profile. A multidisciplinary approach and the use of neuromonitoring contribute to the technical success of surgery; however, careful case selection is essential in order to adequately balance clinical benefits and surgical invasiveness. This complex approach requires a multidisciplinary team and has a steep learning curve.

## Figures and Tables

**Table 1:** Patient characteristics.

Case	Gender	Age (Years)	Disease Treated	Index Level	ASA Score	SINS	Preoperative Symptoms
1	M	42	Metastasis (tongue squamous cell carcinoma)	T1	3	16	Bilateral C8 Syndrome
2	M	49	Traumatic Fracture Type-B3 (AO)	C7&T1	2	11	Axial loading pain
3	M	55	Metastasis (multiple myeloma)	T3	2	14	Axial loading pain
4	F	43	Metastasis (invasive breast cancer)	T1	3	16	Left-sided C8 hypoesthesia
5	F	74	Disc herniation	C7/T1	2	9	Sensitive cross-section from Th3 dermatome
6	M	22	Tuberculosis	T1	2	15	Axial loading pain
7	M	51	Pyogenous Spondylodiscitis	C6-T1	2	12	Axial loading pain
8	M	66	Metastasis (Non-small cell lung cancer)	T1	2	16	Axial loading pain and right-sided hemiparesis
**Median (range)**	**75% M** **25% F**	**49.9** **(22–74)**			**2.0** **(2–3)**	**14.5** **(9–16)**	

**M** = Male; **F** = Female; **ASA Score**: American Society of Anesthesiologists Score; **SINS**: Spinal Instability Neoplastic Score; **AO**: Arbeitsgemeinschaft Osteosynthese Classification of Subaxial Spine Fractures.

**Table 2:** Surgical outcome.

Case	Index Level	Duration of Surgery (Minutes)	Anterior Plating	Posterior Instrumentation	Blood Loss (mL)	Postoperative Complications	ICU Stay (Days)	Hospital Stay (Days)	Follow-Up (Months)
1	T1	255	Yes	No	300		1	14	2.2
2	C7 and T1	293 **	Yes	Yes C5-T3	600 *	Posterior wound-healing disorder	6	6	32.9
3	T3	515 **	No	Yes C7-T6	1700 *	Dysphagia, transient laryngeal nerve dysfunction	1	16	45.7
4	T1	287	Yes	No	200		1	8	5.1
5	C7/T1	280	Yes	No	200		0	6	2.7
6	T1	241	Yes	No	1000	Dysphagia, transient laryngeal nerve dysfunction	0	9	2.5
7	C6-T1	396 **	No	Yes C2-T3	500 *		20	20	1
8	T1	310 **	No	Yes C4-T2	1200 *	Posterior wound-healing disorder	2	15	2.3
**Median (range)**		**290** **(241–515)**			**550** **(200–1700)**		**1** **(1–20)**	**11.5** **(6–20)**	**2.6** **(1–45.7)**

ICU: intensive care unit. * Patients requiring blood transfusion. ** Duration of surgery given for anterior procedure only.

## Data Availability

The data presented in this study are available on request from the corresponding author. The data are not publicly available due to contained information compromising privacy necessitating informed consent.
